# Supplementary material for: The median effective concentration of ropivacaine for ultrasound-guided anterior iliopsoas muscle space block in the elderly undergoing hip surgery: a dose-finding study
Source: PeerJ. 2024 Aug 28;12:e17970. doi: 10.7717/peerj.17970 (PMC11365473; doi:10.7717/peerj.17970)
Supplement: Supplemental Information 3 [file peerj-12-17970-s003.doc]

**研究方案**

研究名称：前路髂腰肌间隙阻滞预防性镇痛对老年髋部骨折患者应激反应及术后认知功能的影响

研究来源：自选

主要研究者/所在科室：马鹏/麻醉科

1. **研究背景**

随着我国社会老龄化程度日益加剧,罹患髋部骨折的患者也在逐年增加。老年患者由于骨质疏松使其更容易发生骨折，髋部骨折又因其并发症多且有较高的围术期死亡率，被称为老年患者的“最后一次骨折”。老年患者因其抵抗风险及耐受手术的能力有限，住院期间并发症发生率高，尤其是术后认知功能障碍（Postoperative cognitive dysfunction,POCD）。POCD的发生将延长老年髋部骨折患者的住院时间，增加患者围术期死亡率及术后发生长期认知功能障碍的风险。髋部骨折后最初24小时内剧烈疼痛的发生率为50%-70%。术前控制不佳的重度疼痛易加重老年患者的认知负担导致POCD。因此，术前有效镇痛治疗是减轻患者疼痛及预防POCD一项重要内容。

对于围术期的患者而言，术前的镇痛治疗较少被关注且镇痛不全的情况也时有发生。髋部骨折患者在创伤和骨折后即引起了强烈的疼痛及应激反应，这将增加老年患者发生POCD的风险，并且许多患者在术前就已经发生了认知功能障碍。有研究表明随着术前疼痛强度的增加，发生POCD的风险也随之增加。老年患者对疼痛的感知程度具有明显的个体差异且对疼痛的耐受性远不如年轻人；同时老年患者痛觉下行调制系统减退，对中、重度的疼痛的反应增强；如不能进行有效的镇痛治疗，疼痛在向中枢的传递过程中伴随应激反应的发生以促使机体抵御外界有害刺激，在该过程中主要涉及下丘脑-垂体-肾上腺轴(HPA axis)和交感系统，增加肾上腺皮质激素、肾上腺髓质激素及甲状腺激素等激素的分泌，最终导致机体代谢及氧耗增加;有研究表明老年患者术后糖皮质激素水平与POCD程度和POCD风险相关。另外，急性疼痛也增加了疼痛转化为慢性疼痛的风险。因此，对于髋部骨折老年患者的镇痛治疗而言，其应包含围术期各个阶段。其次，老年患者镇痛药物及方法的选择较成年人被动。临床上一般使用阿片类药物和非甾体抗炎药（NSAIDS）镇痛，阿片类药物可以很好的缓解围术期急性中、重度疼痛，但其可以引起恶心和呕吐、便秘、瘙痒、镇静、呼吸抑制等不良反应；且阿片类药物用于老年髋部骨折患者术前镇痛的效果还有待明确。NSAIDs因其副作用少于阿片类镇痛药且疗效可，在临床上应用较为广泛，但研究表明NSAIDs对急性重度疼痛疗效有限。目前临床常用硬膜外镇痛虽给患者带来了良好的镇痛效果，但髋部骨折患者入院后常规予肝素进行抗凝治疗，需警惕硬膜外血肿的风险。另外，硬膜外镇痛用于髋部骨折患者的术前镇痛治疗还需要大量的研究去论证。

近年来，随着超声技术定位神经的不断发展，B超引导下单次神经阻滞、目标神经周围置管行神经阻滞已成为围术期卓有成效的镇痛手段。已有很多研究将B超引导下神经阻滞用于术前创伤患者的镇痛，其效果十分确切。但髋部神经支配复杂，目前还没有一个既操作简单又安全有效的神经阻方法，理论上通过局麻药在髂腰肌间隙的扩散，可阻滞股神经和闭孔神经，并部分阻滞股外侧皮神经，给髋部骨折患者提供良好的镇痛。通过前路法阻滞髂腰肌间隙距离内脏器官较远，不会产生损伤风险。且前路髂腰肌间隙阻滞是在患者平卧位时实施，可以使患者避免因体位变化引起疼痛加剧、血流动力学波动及其产生的一系列并发症。因此，本研究将探讨前路髂腰肌间隙阻滞预防性镇痛是否能更有效的缓解围术期疼痛，降低POCD的发生率，为临床上老年髋部骨折患者镇痛方法的选择、POCD的预防提供新的思路。

**二、研究目的**

本研究拟首先评估注入尸体标本前路髂腰肌间隙的亚甲蓝扩散情况，为临床试验提供解剖学依据，同时研究盐酸罗哌卡因应用于老年髋部骨折患者前路髂腰肌间隙阻滞时的90%最低有效浓度和90%最低有效容量，指导临床实践。最后通过观察术前前路髂腰肌间隙阻滞对老年髋部骨折患者围术期疼痛、 应激反应及POCD发生率的影响，探讨该种镇痛方式在减轻患者疼痛的同时是否可以减轻患者应激反应及POCD的发生。

**三、研究设计与试验步骤**

**1. 尸体模型上前路髂腰肌间隙染色后的扩散范围观察**

**1.1 尸体标本的处理**

本研究共收集5具尸体模型（10个标本），标本模型均按照标准操作程序经过甲醛甘油酒精溶液浸泡处理，符合江苏大学医学院解剖学实验室管理制度和道德伦理标准，5具尸体标本与供体相对应，男女随机，编号 A、B、C、D、E，无髋部畸形、外伤及肿瘤史，髋部皮肤完整。

**1.2 超声引导下标本上的前路髂腰肌间隙阻滞**

将尸体标本仰卧位置于解剖实验台上，使用索诺声便携式超声仪，在髂前上棘下方靠内侧3-4 厘米处放置2-5MHz凸阵探头，进行横截面扫描。股外侧皮神经从腰大肌侧面走行，经髂筋膜内斜行穿过髂肌前侧面；股神经从腰大肌后部下降，在 L5 或 S1 水平肌肉外侧边界出现，于腰大肌髂肌夹角，经髂筋膜内下行离开骨盆；闭孔神经从腰大肌后部下降，在 L5水平肌肉内侧边界出现；腰骶干位于腰大肌内侧。于超声探头外侧进针，行平面内阻滞，将20ml 1%亚甲蓝注射至髂腰肌间隙内。

**1.3 尸体标本的解剖及神经染色范围观察**

2h后首先作两处贯穿肌肉全层切口：第一切口从腹股沟浅环旁开三指起，平行于腹股沟韧带向头侧延伸，终止于髂前上棘侧上方。第二个切口从髂前上棘上方倾斜延伸，大致沿着缝匠肌到达大腿内侧。解剖出的组织瓣被抬高并向内翻卷，以暴露髂腰肌的边界。屈曲髋关节，使髂腰肌抬起，以便在髂腰肌间隙中识别股神经、闭孔神经、股外侧皮神经。部分标本髂腰肌被切断，以获得更好的记录视野。暴露出染料的着色区域，记录并描述其扩散范围。

**1.4研究并记录数据**

研究记录的数据为：（1）股神经染色率；（2）闭孔神经的染色率；（3）股外侧皮神经的染色率；（4）从皮肤到目标前路髂腰肌间隙的距离；（5）针头轨迹的长度。

**2.** **盐酸罗哌卡因用于超声引导下前路髂腰肌间隙阻滞最低有效浓度/容量研究**

**2.1盐酸罗哌卡因用于超声引导下前路髂腰肌间隙阻滞90%最低有效浓度研究**

**2.1.1 研究对象** 研究方案需经我院临床伦理委员会审核批准。所有病例选择均将履行知情告知程序，符合自愿参与原则，并签署知情同意书。本研究采用前瞻性队列研究方法，选择在江苏大学附属医院手 术室行髋部骨折择期手的患者，性别不限。 纳入标准：1）ASA分级Ⅱ～Ⅲ级；2）年龄≥65岁；3）BMI18.5～27.9kg/m^2^；4）自愿参与研究。排除标准：1）拒绝参加研究 ；2）对局部麻醉药过敏； 3）穿刺部位感染；4） 合并外周神经疾病；5）髋部既往外伤、手术史；6）合并严重系统性疾病；7）智力障碍且无法沟通。

**2.1.2 麻醉方法** 患者常规监护心电图、上肢无创血压、脉搏氧饱和度，通过鼻导管给氧4L/min，非手术侧上肢建立一条18G静脉通道，静滴林格液维持。患者取平卧位，穿刺部位皮肤使用碘伏消毒，在髂前上棘下方靠内侧3-4厘米处放置 2-5MHz 凸阵探头，进行横截面扫描。股外侧皮神经从腰大肌侧面走行，经髂筋膜内斜行穿过髂肌前侧面；股神经从腰大肌后部下降，在L5或S1水平肌肉外侧边界出现，于腰大肌髂肌夹角，经髂筋膜内下行离开骨盆；闭孔神经从腰大肌后部下降，在L5水平肌肉 内侧边界出现；腰骶干位于腰大肌内侧。于超声探头外侧进针,行平面内阻滞，负吸后无血将25ml盐酸罗哌卡因注射至髂腰肌间隙内，再使用超声扫描，观察局麻药扩散情况。

**2.1.3 数据收集** 从神经阻滞完成开始计时，由独立的评估者（对所使用的罗哌卡因浓度不知情）在第10、20 、30分钟时各测试一次麻醉果并记录，由第30分钟时的效果判断该次阻滞是否成功。评估者用锐利的25G针头对股神经、闭孔神经、股外侧皮神经支配区域皮肤感觉阻滞倩况逐一进行测试。各部位阻滞效果的评价标准为：2分＝无感觉，1分＝ 感觉,减退，0分＝与对侧比较感觉相 同。 只有当总分为6分才认为此阻滞效果为“成功”（阳性反应）， 否则认为阻滞效果为“失败”（阴性反应）。评估者同时记录阻滞过程中异感、血管损伤等并 发症发生情况，并在术后一周随访患者以询问是否存在持续的感觉异常、局部肢 体无力等神经损伤的症状。

2.1.4 **统计学分析** 本研究采用改良Dixon上下序贯法，除第一位患者使用的罗哌卡因浓度为0.2%外，接下来每一位患者接受神经阻滞的局麻药浓度都取决于前一位患者的阻滞效果。 如果前一位患者效果为“失败”（阴性反应）， 则该患者所用局麻药浓度増加一个单位（0.05%）； 如果前一位患者效果为“成功”（阳性反应）， 则该患者所用局麻药浓度减小一个单位（0.05%），根据统计学的要求,至少出现7个有效-无效拐点可认为研究有效， 总患者数无法预知。最终使用Ｒ统计软件，采用线性模型、线性对数模型、概率回归和保序回归，计算出MEC_90_。

**2.2盐酸罗哌卡因用于超声引导下前路髂腰肌间隙阻滞90%最低有效容量研究**

**2.2.1研究对象** 研究方案需经我院临床伦理委员会审核批准。所有病例选择均将履行知情告知程序，符合自愿参与原则，并签署知情同意书。本研究采用前瞻性队列研究方法，根据PASS软件计算样本量，选择在江苏大学附属医院手术室行髋部骨折择期手的患者，性别不限。 纳入标准：1）ASA分级Ⅱ～Ⅲ级；2）年龄≥65岁；3）BMI18.5～27.9kg/m2；4）自愿参与研究。排除标准：1）拒绝参加研究 ；2)对局部麻醉药过敏；3）穿剌部位感染； 4）合并外周神经疾病；5）髋部既往外伤、手术史；6）合并严重系统性疾病；7）智力障碍且无法沟通。

**2.2.2麻醉方法** 患者常规监护心电图、上肢无创血压、脉搏氧饱和度，通过鼻导管给氧4L/min，非手术侧上肢建立一条18G静脉通道，静滴林格液维持。患者取平卧位，穿刺部位皮肤使用碘伏消毒，在髂前上棘下方靠内侧3-4厘米处放置 2-5MHz 凸阵探头，进行横截面扫描。股外侧皮神经从腰大肌侧面走行，经髂筋膜内斜行穿过髂肌前侧面；股神经从腰大肌后部下降，在L5或S1水平肌肉外侧边界出现，于腰大肌髂肌夹角，经髂筋膜内下行离开骨盆；闭孔神经从腰大肌后部下降，在L5水平肌肉 内侧边界出现；腰骶干位于腰大肌内侧。于超声探头外侧进针，行平面内阻滞，负吸后无血将盐酸罗哌卡因注射至髂腰肌间隙内，再使用超声扫描，观察局麻药扩散情况。

**2.2.3 数据收集** 从神经阻滞完成开始计时，由独立的评估者（对所使用的罗哌卡因浓度不知情）在第10、20 、30分钟时各测试一次麻醉效果并记录，由第30分钟时的效果判断该次阻滞是否成功。评估者用锐利的25G针头对股神经、闭孔神经、股外侧皮神经支配区域皮肤感觉阻滞情况逐一进行测试。各部位阻滞效果的评价标准为：2分＝无感觉，1分＝感觉,减退，0分＝与对侧比较感觉相同。 只有当总分为6分才认为此阻滞效果为“成功”（阳性反应）， 否则认为阻滞效果为“失败”（阴性反应）。评估者同时记录阻滞过程中异感、血管损伤等并 发症发生情况，并在术后一周随访患者以询问是否存在持续的感觉异常、局部肢 体无力等神经损伤的症状。

2.2.4 **统计学分析** 本研究采用采用改良Dixon上下序贯法，所有入组患者使用MEC_90_罗哌卡因，除第一位患者使用的罗哌卡因容量为20ml外，接下来每一位患者接受神经阻滞的局麻药容量都取决于前一位患者的阻滞效果。 如果前一位患者效果为“失败”（阴性反应）， 则该患者所用局麻药容量増加一个单位（2.0mL）；如果前一位患者效果为“成功”（阳性反应），则该患者所用局麻药容量减小一个单位（2.0mL），。为得出MEV_90_，根据统计学的要求，至少出现7个有效-无效拐点可认为研究有效，总患者数无法预知。最终使用Ｒ统计软件，通过保序回归,计算出MEV_90_。

**3.前路髂腰肌间隙阻滞预防性镇痛对老年髋部骨折患者应激反应及术后认知功能影响的临床研究**

**3.1 研究对象**

研究方案需经我院临床伦理委员会审核批准。所有病例选择均将履行知情告知程序，符合自愿参与原则，并签署知情同意书。

**A．入选标准：**

①髋部骨折拟择期手术患者，手术类型包括全髋置换术、半髋置换术、PFNA(股骨近端防旋髓内钉内固定术），美国麻醉医师协会(ASA)分级Ⅱ～Ⅲ级；

②年龄≥65岁，BMI18.5～27.9；

③同意接受预防性镇痛方案；

④自愿参与研究。

**B．排除标准：**

①术前访视发现已有明显认知功能受损MMSE评分文盲＜17分，小学＜20分，初中及以上＜24分)；

②髋部手术史；

③阿片类药物或酒精滥用史；

④慢性疼痛病史；

⑤神经系统疾病；

⑥严重视力障碍；

⑦贫血（Hb＜80g/L）,凝血障碍；

⑧无法与医生进行有效的口头沟通；

⑨中途自愿退出研究；

⑩术前合并心衰、呼吸功能严重降低、严重心律失常，控制不良的高血压病、糖尿病等基础疾病患者。

**3.2 研究方法**

**3.2.1病例分组**

采用随机、单盲的方式，计划征集我院2023年7月～2024年2月间80例因髋部骨折拟择期手术患者，应用随机数字表将患者分为对照组（C组）和预防性镇痛组（Q组），所有手术均由我院骨科医生完成，麻醉方式采用蛛网膜下腔阻滞麻醉。数据采集由经过培训的骨科及麻醉相对固定专业医护人员负责。

**3.2.2 实验方法**

**3.2.2.1对照组（C组）**：入室后监测无创（或有创）血压、脉氧饱和度、心电图，建立静脉通路后，予帕洛诺司琼0.075mg静注，右美托咪定0.3-0.6μg/kg/h静脉泵注维持镇静，术中补液采用乳酸钠林格液及羟乙基淀粉（万汶）。麻醉方式采取蛛网膜下腔阻滞，遇操作困难时采用超声辅助下引导穿刺，遇脑脊液回流后抽取澄明脑脊液3ml,留置标本后注入局麻药；术中体温保护，术后采用静脉自控镇痛：盐酸氢吗啡酮0.1mg/kg加生理盐水配成100 ml，手术结束前30min（或冲洗、缝合切口前）静脉给予20μg/kg负荷量，镇痛泵参数设置为单次剂量3~5μg/Kg,背景剂量2ml/h，锁定时间15min。

具体麻醉方法：

①患者垂直侧卧，患侧在上方，健侧下肢屈膝、头弯，头部垫枕，成“虾米状”；

②确定L3-4腰椎间隙；

③皮肤消毒、铺洞巾，再次确认穿刺点，使用1%利多卡因逐层对皮肤、棘上韧带、棘间韧带做局部浸润；

④采用正中或旁正中法穿刺，穿刺遇有脑脊液回流，连接针筒回抽通畅，缓慢抽取澄明脑脊液3ml，留作测定β-淀粉样蛋白42含量，随后注入0.75%盐酸罗哌卡因12-15mg。

⑤如试穿3次不成功，则在超声引导下辅助穿刺。

**3.2.2.2 预防性镇痛组（Q组）**：完善相关检查后，在手术前一日对患者行单次前路髂腰肌间隙阻滞，余手术麻醉、围术期管理、术后镇痛方案同对照组。

**前路髂腰肌间隙具体操作：**

前路髂腰肌间隙组患者取平卧位，穿刺部位皮肤使用碘伏消毒，在髂前上棘 下方靠内侧3-4厘米处放置2-5MHz凸阵探头，进行横截面扫描。股外侧皮神经从腰大肌侧面走行，经髂筋膜内斜行穿过髂肌前侧面；股神经从腰大肌后部下降，在L5或S1水平肌肉外侧边界出现，于腰大肌髂肌夹角，经髂筋膜内下行离开骨盆；闭孔神经从腰大肌后部下降，在L5水平肌肉 内侧边界出现；腰骶干位于腰大肌内侧。于超声探头外侧进针，行平面内阻滞，负吸后无血将局部麻醉药注射至髂腰肌间隙内。盐酸罗哌卡因的使用浓度和容量分别为MEC_90_ 和MEV_90_。

**3.2.2.3术中处理**：控制血压波动不超过病房内安静状态血压的20%，发生低血压时予麻黄素5-10mg/次或去氧肾上腺素50-100μg/次静注，收缩压>180mmHg时给予乌拉地尔10mg/次；如切皮后镇痛不全则行气管插管全身麻醉，患者剔出研究。

**3.2.2.4术后管理**：两组术后均行PCIA镇痛，镇痛目标为运动VAS评分≤3分，如患者运动VAS评分＞3分或行3次有效按压PCIA仍不能获得满意镇痛，则静注帕瑞昔布钠40mg补救，必要时重复给药；术后恶心、呕吐予盐酸帕洛诺司琼0.075mg补救一次，如无改善则暂停使用PCIA。

**3.2.3 监测指标**

**3.2.3.1 一般资料** 记录患者的性别、年龄、ASA 分级、BMI 指数、入院时 MMSE 评分、手术方式、手术时间、术中出血量及住院时间。

**3.2.3.2疼痛评估** 采用视觉模拟评分法（VAS）评估患者疼痛情况：记录两组患者入院时（T1）、手术前1天行镇痛治疗后 1h（T2）、手术日清晨（T3）、椎管内麻醉摆放体位时（T4）、手术后1天清晨（T5）、手术后2天清晨（T6）时患者静息及运动状态下的（运动状态是指将患肢抬离床面 15°） VAS评分。

**3.2.3.3 应激反应评估**

（1）分别记录患者在 T1、T2、T3、T4、T5、T6 时点的心率及平均动脉压。

（2）所有患者在 T1、T3 及 T5 这三个时点抽取静脉血。收集后如不能及时 离心暂冷藏于4℃的冰箱中。收集后及时将血样离心（4℃，3000r/min，10min）， 离心后取上层血浆于 EP 管中，封存于-80℃冰箱，待标本收集完成后用化学免疫 发光法检测血浆糖皮质激素浓度。

**3.2.3.4 睡眠评分**

采用阿森斯失眠量表,采集术前1天、术后1天、2天、3天的睡眠质量评分；

**3.2.3.5 术后认知功能评估**

采用CAM 量表评估两组患者围术期谵妄发生情况：于患者手术当天、术后 1d、 3d、7d 观察并记录患者认知功能障碍的发生情况。

**3.2.3.6** **术后认知功能障碍相关指标检测**

术前1天、术后3天、7天静脉采血，用实时定量PCR法测定血清miR-132水平，白介素-1、6及神经元特异性烯醇化酶含量，测定脑脊液、血清β-淀粉样蛋白-42含量。

**3.2.3.5镇痛相关不良反应**

随访并记录患者镇痛泵按压次数、围术期不良反应的发生情况如嗜睡、恶心、呕吐等；同时还需观察Q组患者有无局麻药中毒，穿刺部位感染和血肿的情况发生。

**3.3 统计学方法**

采用SPSS 25.0 软件进行数据分析。计量资料用(x±s)表示，组间比较采用独立样本t检验；组内测量的资料各时间点间比较，采用重复测量方差分析、秩和检验；计数资料用n(%)表示，组间比较采用卡方(χ^2^)检验；P＜0.05 表示有统计学意义。

**四、疗效判定**

依据视觉模拟评分表（VAS）进行术后疼痛评分；患者在 T1、T2、T3、T4、T5、T6 时点的心率及平均动脉压；患者T1、T3 及 T5静脉血中血浆糖皮质激素浓度；使用 CAM 量表评估两组患者手术当天、术后 1天、 3天、7天认知功能障碍的发生情况。

**五、数据安全监查**

临床研究将根据风险大小制定相应的数据安全监察计划。所有不良事件均详细记录，恰当处理并追踪直到妥善解决；主要研究者定期对所有不良事件进行累积性回顾，必要时召开研究者会议评估研究的风险与受益。

**六、临床研究的伦理学**

临床研究将遵循WMA《赫尔辛基宣言》（2013）、CIOMS《人体生物医学研究国际道德指南》（2002）、原国家卫生部《涉及人的生物医学研究伦理审查办法》（2016）等相关规定的伦理原则，在研究开始前，由伦理委员会批准该试验方案后才可实施临床研究。每一位受试者入选本研究前，研究者有责任向受试者或其代理人完整、全面地介绍本研究的目的、程序和可能的风险，并签署书面知情同意书，应让受试者知道他们有权随时退出本研究，知情同意书应作为临床研究文件保留备查。研究过程中将保护受试者的个人隐私与数据机密性。

**七、研究进度**

2022年12月-2024年10月：临床试验，收集数据，数据分析，撰写文章
